# Subscapular Abscess Caused by Panton-Valentine Leukocidin-Positive *Staphylococcus aureus*: An Atypical Presentation

**DOI:** 10.1155/2018/8256428

**Published:** 2018-06-10

**Authors:** Kamal Patel, Emma Spowart, Dana Sochorova, Nadia Diego, Georgios Mamarelis, Mohammad Zain Sohail

**Affiliations:** ^1^Accident and Emergency Department, Barnet Hospital, Royal Free London NHS Foundation Trust, Wellhouse Lane, Barnet EN5 3DJ, UK; ^2^Bart's Health NHS Foundation Trust, London, UK; ^3^The Queen Elizabeth Hospital Kings Lynn Foundation Trust, Kings Lynn, Norfolk PE30 4ET, UK; ^4^The Princess Alexandra Hospital NHS Trust, Harlow CM20 1QX, UK; ^5^Royal London Hospital, Whitechapel, London E1 1BB, UK; ^6^East of England Rotation, The Queen Elizabeth Hospital Kings Lynn Foundation Trust, Kings Lynn, Norfolk PE30 4ET, UK

## Abstract

Subscapular abscess is an uncommon condition which requires early recognition followed by prompt surgical intervention. We present a case of spontaneous subscapular abscess following blunt trauma to the shoulder in a patient with a history of recurrent superficial soft tissue infections, in which Panton-Valentine leukocidin-producing *S. aureus* was identified as the infectious agent. This strain due to its virulence can lead to fatal infections in otherwise healthy individuals; therefore, a high index of suspicion is needed to investigate with an MRI to rule out abscess formation in a patient with acute shoulder girdle pain and negative joint aspirate. Urgent surgical intervention and targeted antimicrobial therapy against PVL-positive *S. aureus* in accordance with microbiologist yield good outcomes.

## 1. Introduction

Abscess formation in the area between the subscapularis muscle and the chest wall is an infrequently reported entity. A literature search of PubMed using the terms “subscapular” + “abscess” yields only six relevant case reports [1–6], of which one describes a fatal outcome [2].

Panton-Valentine leukocidin (PVL) is a cytotoxin which causes leukocyte destruction and tissue necrosis. The genes encoding for PVL are present in less than 2% of *S. aureus* species, according to statistics from the UK National Reference Laboratory [7]. Strains of PVL-producing *S. aureus* have been linked to highly virulent and severe community-acquired skin infections and abscesses in otherwise healthy children and young adults [7]. PVL production is seen much more frequently in *S. aureus* strains associated with abscesses or deep-seated soft-tissue infection compared with asymptomatic carriage strains [8].

We present a case involving an active adult woman with no predisposing comorbidities who had experienced a number of superficial skin infections in the preceding year and developed an abscess in the subscapular space following trauma, cultures from which grew PVL-producing *S. aureus*.

## 2. Case Presentation

A fit and active 38-year-old female presented to the Accident and Emergency Department with a four-day history of worsening right shoulder pain radiating down the right arm, with swelling around the shoulder. This was accompanied by intermittent fevers for the preceding two days. The patient graded the pain to be 8/10 on a visual analogue scale for pain. The patient reported an episode of right shoulder pain three weeks prior to current presentation which developed while she was boxing with a punch bag and resolved spontaneously in 2-3 days without seeking any medical advice.

The patient denied any history of infections in the previous 6 weeks. She had a significant past medical history of cellulitis around the leg 6 months prior and a Bartholin cyst that was treated conservatively 8 months before this presentation. She was not on any routine medications and did not have any predisposing medical conditions such as immunosuppression or diabetes.

At presentation, all her observations were essentially unremarkable except temperature which was recorded to be 38.6°C. On examination, the right shoulder was tender and swollen with severely restricted active and passive range of movements. No cellulitis, erythema, or differential warmth was noted.

Haematological investigations showed mild leukocytosis with a white cell count of 11.1 × 10^9^/L with predominant neutrophilia and a C-reactive protein (CRP) level of 233 mg/L. Liver functions tests, urea and electrolytes, bone profile, and coagulation studies were all within normal limits. Plain radiographs of the chest and shoulder were essentially unremarkable. Shoulder aspirate analysis was negative for any organisms, however showed some scanty pus cells. The patient was started on IV flucloxacillin 1 g intravenous four times a day as she was continuing to have temperature spikes, although shoulder aspirate cultures and blood cultures were negative.

**Figure 1:**
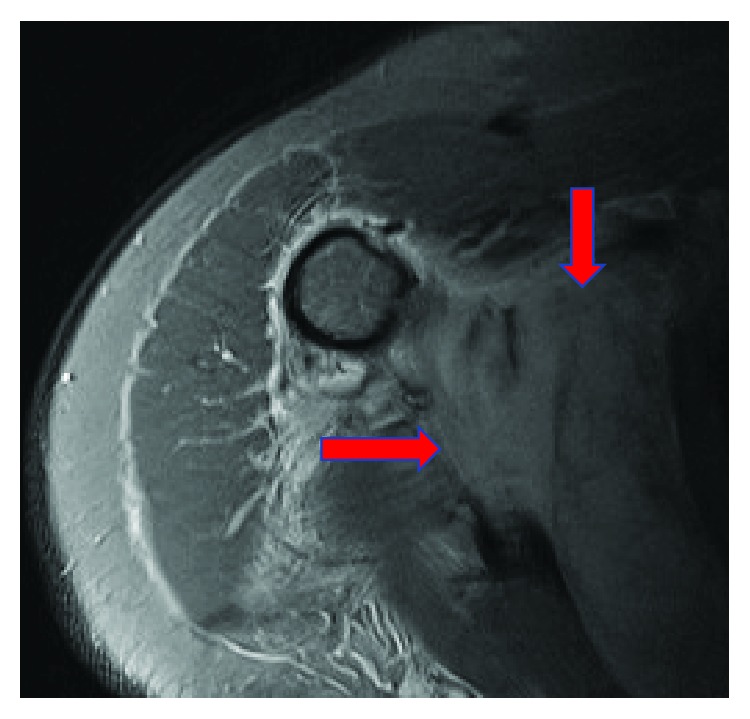
MRI of Rt. shoulder axial STIR sequence demonstrating well-defined ovoid area of hyperintensity.

**Figure 2:**
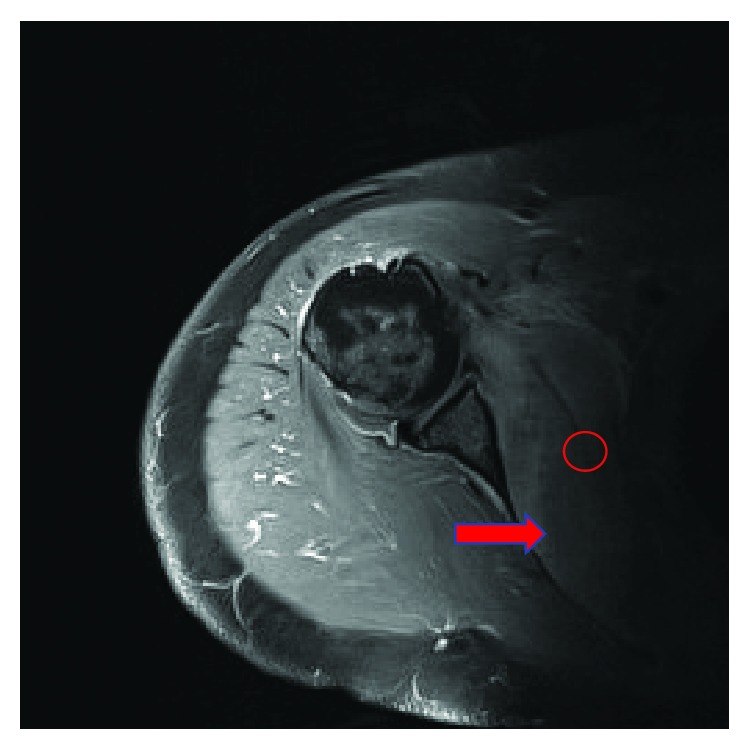
MRI of Rt. shoulder coronal T1 sequence demonstrating odematomous subscapularis muscle.

**Figure 3:**
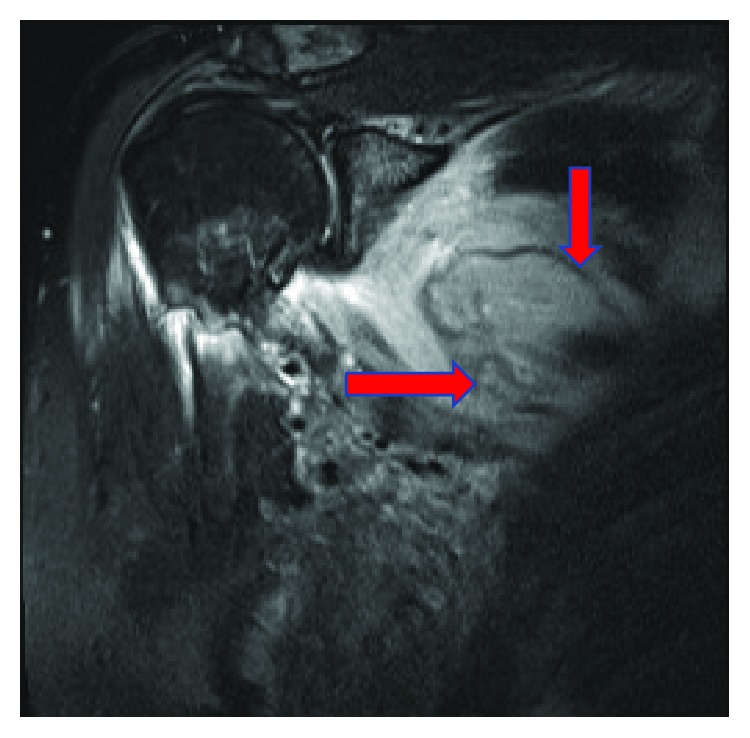
MRI of Rt. shoulder coronal T2 sequence demonstrating well-defined ovoid area of hyperintensity and abscess formation.

Due to the patient's severe symptoms and markedly elevated CRP level, urgent magnetic resonance imaging (MRI) of the right shoulder was performed. This revealed marked oedema throughout the subscapularis muscle with a relatively well-defined ovoid area of hyperintensity on short-tau-inversion-recovery (STIR) (Figure 1) and isointensity to muscle on T1 (Figure 2). The area measured 9 cm on the oblique axial diameter, almost 3 cm in depth, and over 3.5 cm craniocaudally, with fluid extending inferiorly from the subscapular region overlying the chest wall axially measuring over 5 cm transversely and 1.5 cm in depth on T2-weighted images (Figure 3). This MRI confirmed abscess formation within the subscapularis muscle as the cause of the presentation.

The patient underwent surgical open drainage of the right subscapularis abscess under general anaesthesia via a standard deltopectoral approach. During mobilisation of the conjoined tendon, approximately 150 mL of blood-stained pus exuded from the subscapularis muscle. The subscapularis muscle was left with a defect but subscapularis tendon integrity was maintained. Following irrigation, the wound was closed. Cultures of the evacuated pus grew PVL-positive *S. aureus*, sensitive to flucloxacillin. No per operative signs of intraarticular infection were found, and an on table aspirate yielded no organisms on gram stain and cultures. The case was discussed with musculoskeletal microbiologist, and the patient was given a further two-week course of flucloxacillin.

At the 6-week follow-up to assess improvement, the patient's wound had healed well and shoulder pain had resolved with no signs of recurrence of the infection. She still had some restriction in the movement of her shoulder for which she was referred to physiotherapy.

## 3. Discussion

Subscapular abscess is a rare occurrence where a collection of pus forms between the subscapularis muscle and the chest wall. Presentation with signs of underlying sepsis along with a focus of symptoms around the shoulder in a generally healthy patient necessitated thorough investigation. In our patient, the diagnosis was made following an urgent MRI scan with a high index of suspicion for an abnormality within the shoulder girdle.

In this case, the abscess is likely to have formed following trauma to the shoulder girdle while boxing, leading to a subscapular haematoma. Previous blunt trauma was proven to play a key role in abscess formation as demonstrated in the case report of a 7-year-old boy with subscapular abscess following a trauma [3] and the case of a 19-year-old man where subscapular abscess with subsequent severe pneumonia had a fatal outcome in a previously healthy young individual [2]. As shown above, the history of trauma should always point to abscess as one of the important differential diagnosis.

Previous history of skin or soft tissue infection should always raise the suspicion of PVL-producing strains despite well-resolved superficial infection as well documented by cases mentioned above. Our patient was previously treated for cellulitis and Bartholin cyst. The literature demonstrates a case of a 23-year-old patient who was readmitted shortly after incision and drainage of a Bartholin's cyst with sepsis requiring admission to intensive care. CT scan revealed multiple abscesses in the pectoralis, supraspinatus, and gluteus muscles; however, vaginal examination showed no signs of ongoing infection at the site of drainage [9]. Relatively benign skin or soft tissue infections should always be considered as a significant risk factor for further haematological spread with possible life-threatening conditions. It is possible that the patient in our case either had a much longer latent phase than previously reported for haematological spread or that she had a minor, insignificant-seeming soft tissue or skin injury that was overlooked on this presentation.

As PVL gene detection is not routinely tested for, samples must usually be submitted to a national laboratory [10]. PVL should be suspected and tested for in all cases of recurrent soft tissue infections, especially those with recurrent skin infections, to enable antibiotic optimisation to prevent further haematogenic spread and to prevent potentially severe necrotising haemorrhagic pneumonia. Pneumonia caused by PVL-producing *S. aureus* affects younger and healthier individuals compared to non-PVL-producing *S. aureus* [11, 12] and can have a fulminant clinical picture with fatal outcomes in up to 75% of cases [13]. The significance of the role of PVL regarding invasiveness and worse outcomes is potentially different in skin and soft tissue infection compared to lung and bone involvement [14]. However, small study groups of osteomyelitis and arthritis caused by *Staphylococcus* strains producing PVL in paediatric populations showed a more dramatic picture of infection and prolonged treatment [15].

The treatment for any infection of this type involves prompt drainage of the abscess. In this case, antibiotic treatment was also indicated due to the raised CRP [16]. To enable rationalisation of antibiotics and prevent spread of resistant strains, samples of fluid obtained when performing drainage should be sent off for culture and microscopy as standard practice [17]. Empirical antibiotic therapy for mild forms of skin and soft tissue infections includes flucloxacillin and clindamycin as the antibiotics of choice. When severe, deep soft tissue PVL-SA (MSSA or MRSA) infections are suspected, parenteral vancomycin, teicoplanin, daptomycin, or linezolid are recommended [8]. Individual treatment should be guided by antimicrobial susceptibilities, and tissue penetration of the antibiotics should be taken into consideration to ensure optimal clinical outcomes [10]. The reported antitoxin effects of clindamycin, linezolid, and rifampicin support the use of these antibiotics in the treatment of PVL-producing *S. aureus* [18].

The guidelines for decolonisation vary between the USA and the UK. To prevent repeated infections and to reduce transmission, current UK guidelines recommend decolonisation regime with mupirocin and chlorhexidine, following completion of treatment for the acute infection [9]. However, there is little evidence for decolonisation or screening for household contacts supported by randomised control trials.

Animal models showed that PVL gene plays a role of a virulence factor [19] and that PVL production significantly contributes to muscle injury in murine models [20]. These findings correlate with the more severe presentations in paediatric patients with infective pyomyositis and myositis with PVL-positive isolates compared to PVL-negative *S. aureus* strains [21, 22].

As there is no consensus on the exact cytolytic role of PVL in relation to severity of infection, both animal modelling and clinical studies will be required to clarify the exact mechanism of tissue injury related to PVL production.

To our knowledge, this is the first reported case of PVL-positive *S. aureus* causing a subscapular abscess. In an otherwise healthy patient with repeated skin infections, PVL-positive *S. aureus* should be considered as a differential alongside with septic arthritis, and eradication therapy offered following treatment. In a patient with joint pain and raised inflammatory markers, abscess should be considered as one of the differentials. As in this case, careful history may reveal an earlier injury causing a haematoma as a source of the abscess. We can expect that cases of deep soft tissue infection will occur more frequently due to almost doubled incidence of *S. aureus* bacteraemia over the last 25 years [8] including the dramatic rise in MRSA cases [23]. A similar trend has been depicted in relation to PVL-positive strains with a twofold increase between 2005 and 2006 [24].

In light of both the increasing incidence of PVL-positive *S. aureus* strains and the potential fulminant picture, awareness should be increased among all health care professionals as minor infections such as skin and soft tissue-related ones can precede much more serious soft tissue infections in young immunocompetent populations. Controlling the spread of PVL-positive *S. aureus* could also help to prevent potentially life-threatening cases of necrotising pneumonia.

The prompt diagnosis and treatment with drainage likely contributed to the good outcome at follow-up in this case.
